# Monoamine Loss and Functional Connectivity Alterations in Patients With Parkinson's Disease and Depression

**DOI:** 10.1002/cns.70705

**Published:** 2025-12-25

**Authors:** Bao‐Lei Xu, Xiu‐Lin Liu, Olivier Barret, Gilles D. Tamagnan, Hong‐Wen Qiao, Jie Lu, Chun Zhang, Piu Chan, Shu‐Ying Liu

**Affiliations:** ^1^ Department of Neurology Xuanwu Hospital, Capital Medical University Beijing China; ^2^ National Clinical Research Center for Geriatric Diseases Beijing China; ^3^ Université Paris‐Saclay, CEA, CNRS, MIRCen, Laboratoire des Maladies Neurodégénératives Fontenay‐aux‐Roses France; ^4^ XingImaging LLC New Haven Connecticut USA; ^5^ Department of Radiology and Nuclear Medicine Xuanwu Hospital, Capital Medical University Beijing China; ^6^ Chinese Institute for Brain Research (CIBR) Beijing China

**Keywords:** ^18^F‐FP‐DTBZ‐PET, depression, monoaminergic deficits, Parkinson's disease, rs‐fMRI

## Abstract

**Background:**

The pathophysiology and neural substrates of depression in Parkinson's disease remain unclear. It has been associated with dysregulation of the monoaminergic systems. Furthermore, functional abnormalities within the mesolimbic‐basal ganglia reward circuit may be involved in the pathogenesis of Parkinson's disease‐related depression.

**Objectives:**

We aimed to investigate monoaminergic deficits and the corresponding changes within the striatum‐mesolimbic circuitry in patients with Parkinson's disease and depression.

**Methods:**

This study included eight depressed and 18 non‐depressed patients with Parkinson's disease and 28 matched healthy controls. We simultaneously obtained ^18^F‐fluoropropyl‐dihydrotetrabenazine positron emission tomography (^18^F‐FP‐DTBZ PET) and resting‐state functional magnetic resonance images (rs‐fMRI). We also recruited additional patients with Parkinson's disease (nine depressed and 20 non‐depressed) for the validation of the rs‐fMRI analysis. The ^18^F‐FP‐DTBZ standardized uptake value ratio relative to the occipital lobe was used to quantify monoaminergic deficits in the striatum‐mesolimbic subregion. Voxel‐ and volume‐of‐interest‐based analyses were performed to investigate the functional connectivity changes in the striatum‐mesolimbic circuitry of depressed patients.

**Results:**

Patients who were depressed exhibited greater monoaminergic deficits in the anterior dorsal and ventral putamen and the nucleus‐accumbens than non‐depressed patients did. Depression severity was negatively correlated with standardized uptake value ratio in the anterior ventral putamen and the nucleus‐accumbens of patients with Parkinson's disease. Compared with non‐depressed patients, higher functional connectivity was noted within the striatum‐mesolimbic circuit in depressed patients.

**Conclusions:**

In depressed Parkinson's disease patients, monoaminergic deficits are present alongside abnormally increased connectivity within the striatum‐mesolimbic circuitry, highlighting this pathway as a potential target for treatment.

## Graphical abstract

PET/MRI evidence links striatum‐mesolimbic monoaminergic loss and functional hyperconnectivity with depression severity in Parkinson's disease. Depressed patients show hyperconnectivity in striatum‐mesolimbic circuits versus non‐depressed and healthy elderlies.

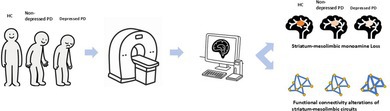

## 1 Introduction

Parkinson's disease, the second most common neurodegenerative disorder [1], is characterized by nigrostriatal dopaminergic neuron loss and phosphorylated α‐synuclein deposition in Lewy bodies and Lewy neurites [2], leading to diverse motor and non‐motor symptoms [3]. Depression, affecting approximately 35% of the patients [4], is one of the earliest and most prevalent non‐motor symptoms across all disease stages, including the prodromal phase [5]. It frequently coexists with anxiety, further worsening quality of life and increasing caregiver burden [6]. The exact pathophysiology of Parkinson's disease‐related depression is unclear. Postmortem histopathological staging has revealed complex neurobiological changes underlying the development of Parkinson's disease. Lewy body pathology may spread sequentially and progressively within monoaminergic innervation in the mesolimbic and basal ganglia reward circuits [2, 7], involving the dysregulation of dopaminergic, serotonergic, noradrenergic, and cholinergic neurotransmitters [8]. Dopamine agonists, selective serotonin reuptake inhibitors, and norepinephrine reuptake inhibitors are used as antidepressant treatments [9]. Therapies such as monoamine oxidase type B inhibitors can alleviate Parkinson's disease‐related depressive symptoms by non‐selectively increasing monoaminergic levels (primarily dopamine) and potentially indirectly modulating serotonergic activity [10]. Neither pharmacological nor non‐pharmacological treatments have exhibited sufficient efficacy, given the high prevalence and heterogeneity of Parkinson's disease‐related depression [11]. Encouragingly, advancements in neuroimaging, including single‐photon emission computed tomography, positron emission tomography (PET), structural magnetic resonance imaging (MRI), and resting‐state functional MRI (rs‐fMRI), offer promising avenues for exploring the pathogenesis of Parkinson's disease‐related depression.

The vesicular monoamine transporter 2 (VMAT2) is crucial for packaging monoamines into presynaptic vesicles [12], and its inhibition can induce depression [13], underscoring the role of monoaminergic dysfunction in mood disorders. Most studies reported a reduction of monoaminergic bindings in the striatum‐mesolimbic regions in PD patients with depression [14, 15, 16, 17], and alterations of serotonin binding in the striatum, anterior cingulate, and raphe nuclei were reported to link with the severity of depression in PD [18], while one reported a lack of association between monoaminergic deficit and depression [19]. However, the underlying mechanism between monoaminergic disruption and depression in Parkinson's disease remains unclear. Previous rs‐fMRI studies had suggested neural network changes within the limbic circuits, primarily involving the limbic system (including the nucleus‐accumbens, amygdala, hippocampus, insula, and cingulate cortex), basal ganglia, and prefrontal cortex, may be associated with depression in Parkinson's disease [20, 21, 22].

Therefore, we hypothesized that localized monoaminergic denervation, particularly within the striatum‐mesolimbic network, may affect the integrity of the corresponding functional connectivity and contribute to the pathophysiology of Parkinson's disease‐related depression. To test this, we used ^18^F‐FP‐DTBZ PET imaging, a technique known for its high sensitivity, specificity, and affinity for VMAT2, to visualize and quantify monoaminergic denervation [23] and simultaneous fMRI, a widely used measurement of spontaneous blood‐oxygen‐level‐dependent neural activity, to assess disruptions in depression‐related brain networks at rest. Utilizing PET/MRI, a hybrid technique that simultaneously assesses brain structure, intrinsic activity, and monoaminergic neurotransmitter transport with optimal spatial and temporal alignment, we investigated how alterations in monoaminergic neurotransmitter distribution and changes in functional connectivity within the striatal and mesolimbic networks are associated with Parkinson's disease‐related depression.

## 2 Methods

### 2.1 Participants and Clinical Evaluation

**FIGURE 1:**
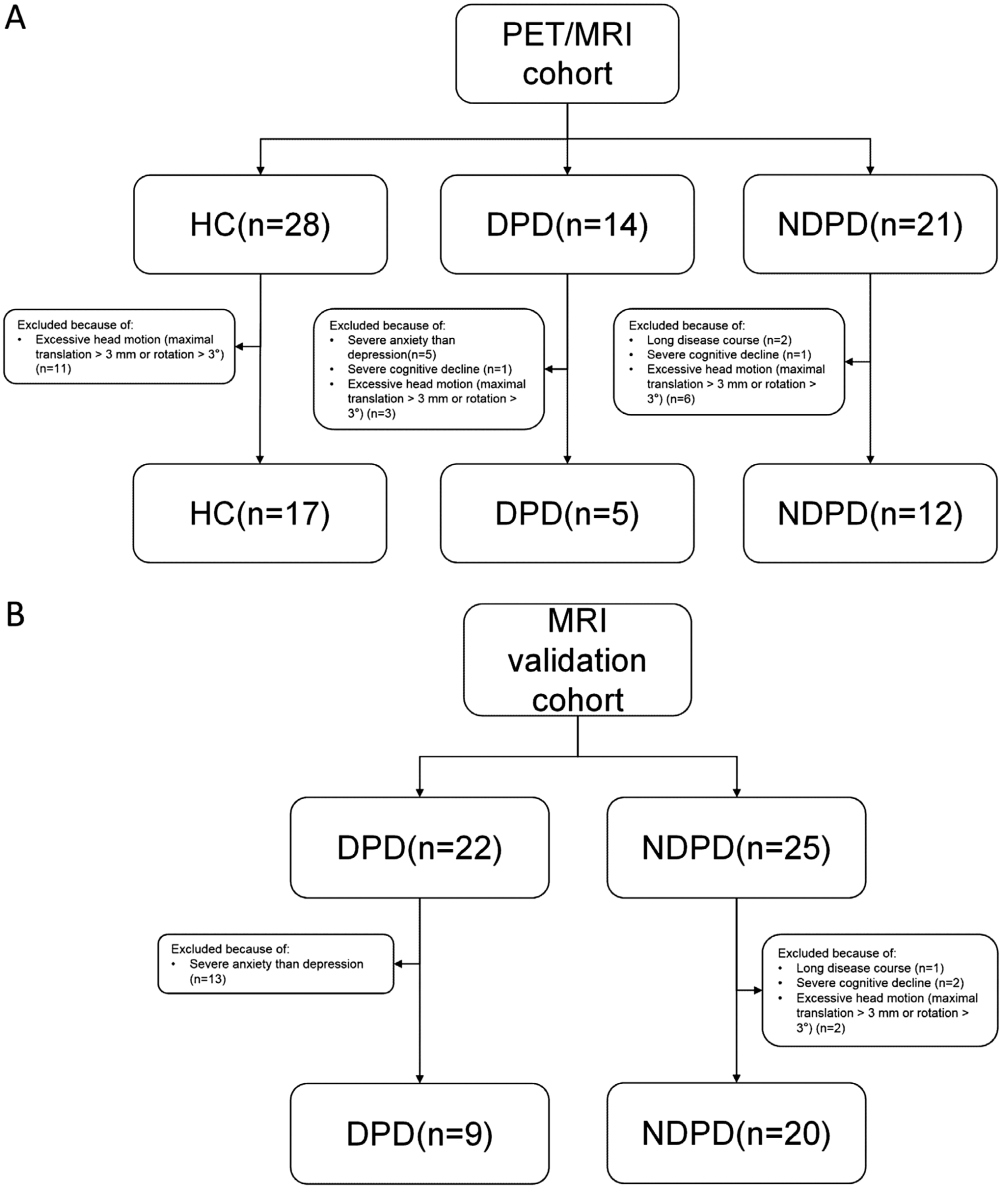
Study cohort enrollment flowchart. HC, healthy control; DPD, Parkinson's disease patients with depression; NDPD, Parkinson's disease patients without depression.

Eighty‐two participants with Parkinson's disease, diagnosed by movement disorder specialists per Movement Disorder Society Clinical Diagnostic Criteria, and 28 age‐/sex‐matched healthy controls were enrolled (2019–2021). Thirty‐five patients with Parkinson's disease and 28 healthy controls comprised the PET/MRI cohort, with 47 additional patients with Parkinson's disease in the MRI validation cohort. Exclusion criteria included major psychiatric/neurological disorders, systemic diseases (e.g., diabetes and hypertension), malignancies, MRI contraindications, and antidepressant/psychotropic medication use. Montreal Cognitive Assessment (MoCA), 24‐item Hamilton Depression Rating Scale (HAMD‐24), 17‐item HAMD (HAMD‐17), Hamilton Anxiety Scale (HAMA), and the Rapid Eye Movement Sleep Behavior Disorder Questionnaire Hong Kong (RBDQ‐HK) were used to assess cognitive and psychiatric symptoms, while the Parkinson's Disease Questionnaire (PDQ‐39), the third part of the Movement Disorder Society‐Unified Parkinson's Disease Rating Scale (MDS‐UPDRS III), and the modified Hoehn‐Yahr (mH‐Y) Scale were applied to measure Parkinson's disease‐specific quality of life and severity of motor symptoms. The classification of depression was based on established cutoff scores on the Hamilton Depression Rating Scale. For the PET/MRI cohort, a cutoff of ≥ 9 on the HAMD‐24 was employed based on a previous study indicating a high sensitivity and specificity in identifying depression in PD [24], resulting in 14 DPD and 21 NDPD patients. In the MRI validation cohort, a cutoff of ≥ 7 on the HAMD‐17 was used as suggested [25], yielding 22 DPD and 25 NDPD patients. After excluding 18 patients with predominant anxiety, four with severe cognitive decline, and three with extended disease duration, the PET/MRI cohort comprised 8 DPD and 18 NDPD patients, and the MRI validation cohort comprised 9 DPD and 22 NDPD patients (Figure 1).

All examinations were performed > 12 h after drug withdrawal, with written informed consent and ethical approval from Xuanwu Hospital Ethics Committee.

### 2.2 Scanning Procedures

^18^F‐FP‐DTBZ was synthesized as previously described [26]. PET/MRI was conducted on a 3.0‐T PET/MR scanner (uPMR790; UIH, Shanghai, China) > 12 h after medication withdrawal. Participants were instructed to close their eyes, relax, and stay awake, with foam pads and earplugs used for motion and noise reduction. Following the intravenous injection of approximately 222 MBq of ^18^F‐FP‐DTBZ and a 90‐min rest, imaging acquisition commenced with previously described acquisition parameters [27]. High‐resolution 3D T1‐weighted images were acquired for attenuation correction, localization, and normalization (field of view = 300 mm, voxel size = 1.82 × 1.82 × 2.78 mm^3^). Rs‐fMRIs were collected using echoplanar imaging (repetition time = 2000 ms, echo time = 30 ms, slices = 31, matrix = 64 × 64, voxel size = 3 × 3 × 3 mm^3^, flip angle = 90°).

MRI was conducted for 31 patients with Parkinson's disease, adhering to the identical scanning workflow and using a 3.0‐T MRI scanner (Siemens, Germany). Axial T1 images were acquired (field of view = 256 mm, voxel size = 1 × 1 × 1 mm^3^) for attenuation correction and spatial normalization. Rs‐fMRIs were collected with gradient‐recalled echo‐planar imaging (repetition time = 2000 ms, echo time = 30 ms, slices = 35, matrix = 64 × 64, voxel size = 3 × 3 × 3 mm^3^, flip angle = 90°).

### 2.3 Image Analysis

#### 2.3.1 PET Data Processing

PMOD (version 4.105) was used for PET image processing and analysis. Each PET image was co‐registered with the corresponding T1 MR image, and each T1 MR image was spatially normalized to the Montreal Neurological Institute (MNI) MR imaging template. Spatial normalization parameters were applied to PET images to generate a spatially normalized PET image in the MNI domain. Based on our existing anatomical segmentation and iterative delineation methods, the dorsal‐raphe, median‐raphe, substantia‐nigra, caudate, anterior‐dorsal‐putamen, anterior‐ventral‐putamen, posterior‐dorsal‐putamen, posterior‐ventral‐putamen, nucleus‐accumbens, and amygdala were defined as volumes of interest (VOIs) [27]. The standardized uptake value ratio (SUVR) of the target VOI was calculated using the occipital cortex as a reference region, with ipsilateral (I) VOIs defined as those on the symptom onset side, and contralateral (C) on the opposite side.

#### 2.3.2 MRI Preprocessing and Functional Connectivity Calculation

RESTplus (V1.28; SPM12/MATLAB 2022b) was used for rs‐fMRI preprocessing. After slice timing and realignment, three DPDs, six NDPDs, and 11 healthy controls from the PET/MRI cohort and two NDPDs from the MRI validation cohort were excluded due to excessive head motion (> 3 mm translation or > 3° rotation). The remaining participants (PET/MRI: 5 DPD, 12 NDPD, 17 healthy controls; MRI validation: 9 DPD, 20 NDPD) underwent reprocessing using FSL‐based image flipping to align left/right hemispheres with ipsilateral/contralateral sides, and this was followed by repeated slice timing and realignment. Subsequent steps included co‐registration to structural images, MNI space normalization, linear detrending (to remove scanner/participant drift), and bandpass filtering (0.01 to 0.08 Hz). Nuisance regression covariates comprised head motion, white matter, and cerebrospinal fluid signals.

Given the roles of the nucleus‐accumbens, striatum, amygdala, hippocampus, and anterior‐cingulate‐cortex in emotion regulation and reward processing [7, 16], a subset of VOIs (bilateral caudate, anterior‐dorsal‐putamen, anterior‐ventral‐putamen, posterior‐dorsal‐putamen, posterior‐ventral‐putamen, nucleus‐accumbens, hippocampus, amygdala, and anterior‐cingulate‐cortex) was selected for rs‐fMRI analyses to evaluate the disruption of striatal monoaminergic terminals in emotion‐related brain circuits. Masks were created and modified using Hammers adult atlases (30 participants, 95 regions) [27, 28]. RESTplus (V1.28) generated voxel‐ and VOI‐based functional connectivity. Seed regions (I‐anterior‐dorsal‐putamen, I‐anterior‐ventral‐putamen, C‐anterior‐ventral‐putamen, I‐nucleus‐accumbens, and C‐nucleus‐accumbens), identified by significant ^18^F‐FP‐DTBZ SUVR differences in DPD versus NDPD, guided voxel‐wise functional connectivity within an 18‐region mask. Fisher's *z*‐transformed Pearson correlations improved normality. Finally, an 18 × 18 symmetric functional connectivity matrix for the striatal‐mesolimbic network was constructed per participant.

To mitigate scanner/protocol‐induced systematic bias and non‐biological variability, we harmonized VOI‐wise functional connectivity matrices (Pearson correlation‐derived) utilizing ComBat (https://github.com/Jfortin1/ComBatHarmonization/tree/master/Matlab) [29]. Site, diagnostic group, and Frame‐wise Displacement Mean were incorporated as covariates to preserve biological trends and prevent overcorrection.

### 2.4 Statistical Analysis

Analyses employed SPSS 27.0 and MATLAB 2022b. Group comparisons of clinical data and subregional SUVRs employed one‐way analysis of variance (ANOVA), *t*‐tests, or non‐parametric alternatives (Kruskal–Wallis/Mann–Whitney), with Tukey's Honestly Significant Difference correction for multiple group comparisons. Based on the distribution characteristics of the data, correlations between HAMD‐24 scores and SUVRs (from the regions with significant monoaminergic deficits in the DPD group) were assessed in the PD patients using Spearman's correlation analysis, with partial correlation analysis controlled for disease duration. Significance was established at *p* < 0.05. Rs‐fMRI data were processed using SPM12 and Network‐Based Statistic (NBS, V1.2) in MATLAB 2022b, with age and sex as covariates. Voxel‐wise analyses included (1) ANOVA for multi‐group comparisons, (2) two‐sample *t*‐tests for inter‐group differences, and (3) multiple regression analyses performed in patients with Parkinson's disease to assess the associations with depression and anxiety, controlling for (i) age, sex, and disease duration, and (ii) age, sex, and MDS‐UPDRS III scores. The results from the primary model (i) are reported in the main text (voxel *p* < 0.001, cluster‐level FWE correction *p* < 0.05, > 10 voxels), while the results from the supplementary model (ii) are presented in the Supporting Informations. VOI‐based analyses employed (1) NBS‐ANOVA (50,000 permutations; edge *p* > 0.01, component *p* > 0.05) and (2) two‐sample *t*‐tests performed using custom MATLAB scripts, with post hoc Bonferroni correction.

## 3 Results

### 3.1 Demographic and Clinical Characteristics

Age, sex, and MoCA scores exhibited no significant group differences in the PET/MRI cohort (Table 1). Healthy controls scored significantly lower than patients on MDS‐UPDRS III, RBDQ‐HK, HAMA, and HAMD‐24 (all *p* < 0.05; Table 1). Between DPD and NDPD, disease duration, mH‐Y scale, and MDS‐UPDRS III scores were comparable. However, DPD exhibited more severe depression (HAMD‐24, *p* < 0.001), anxiety (HAMA, *p* < 0.001), sleep disturbance (RBDQ‐HK, *p* = 0.046), and reduced quality of life (PDQ‐39, *p* = 0.018) than did NDPD. The DPD and NDPD groups in the MRI validation cohort exhibited no significant differences in sex, age, disease duration, mH‐Y scale, MDS‐UPDRS III, MoCA, HAMA, RBDQ‐HK, or PDQ‐39. Nevertheless, depression symptoms were significantly more severe in DPD than they were in NDPD (HAMD‐17, *p* < 0.001, Table S1).

**TABLE 1:** Clinical and demographic data of PET/MRI cohort.

	HC (*N* = 28)	NDPD (*N* = 18)	DPD (*N* = 8)	*p* (HC vs. NDPD vs. DPD)	*p* (NDPD vs. DPD)
Age (years)	57.25 ± 10.87 (36–71)	57.72 ± 15.87 (26–86)	59.63 ± 10.41 (40–73)	0.897^a^	/
Gender	14F/14M	6F/12M	4F/4M	0.516^b^	/
Disease duration (m)	/	24.61 ± 10.02 (7–42)	34.63 ± 22.43 (6–47)	/	0.260^c^
mH‐Y scale	/	1.56 ± 0.54 (1.0–2.5)	2.00 ± 0.53 (1.0–2.5)	/	0.065^d^
MDS‐UPDRS III	0.46 ± 1.37 (0–6)	22.22 ± 10.73 (7–45)	22.75 ± 11.76 (7–44)	0.001^b^	0.977^f^
MoCA	25.14 ± 3.83 (14–30)	26.00 ± 3.81 (16–30)	27.13 ± 1.96 (23–29)	0.371^a^	/
HAMD‐24	1.50 ± 2.12 (0–7)	2.17 ± 1.95 (0–6)	14.75 ± 5.99 (9–24)	< 0.001^b^	< 0.001^f^ *
HAMA	2.21 ± 2.44 (0–9)	2.28 ± 2.40 (0–7)	11.50 ± 4.21 (4–17)	< 0.001^a^	< 0.001^e^ *
RBDQ‐HK	4.50 ± 5.73 (0–25)	8.67 ± 10.91 (0–41)	18.50 ± 11.07 (0–30)	0.013^b^	0.046^f^ *
PDQ‐39	/	14.47 ± 11.96 (0–41)	29.14 ± 14.79 (8–50)	/	0.018^c^

*Note:* Data are presented as mean ± SD, with ranges in parentheses.

Abbreviations: DPD, patients with Parkinson's disease and depression; HAMA, Hamilton Anxiety Scale; HAMD‐24, 24‐item Hamilton Depression Rating Scale; HC, healthy controls; MDS‐UPDRS III, the third part of the MDS‐Unified Parkinson's Disease Rating Scale; mH‐Y Scale, modified Hoehn‐Yahr Scale; MoCA, Montreal Cognitive Assessment; NDPD, patients with Parkinson's disease and without depression; PDQ‐39, Parkinson's Disease Questionnaire; RBDQ‐HK, Rapid Eye Movement Sleep Behavior Disorder Questionnaire‐Hong Kong.

^a^ One‐way ANOVA.

^b^ Kruskal–Wallis test.

^c^ Student's *t*‐test.

^d^ Mann–Whitney *U* test.

* *p value is significant after post‐hoc correction (e: survived Turkey’s Honestly Significant Difference test; f: survived correction of multiple group comparisons after Kruskal‐Wallis test).

### 3.2 ^18^F‐FP‐DTBZ PET Uptake in Striatum and Mesolimbic Subregions

**FIGURE 2:**
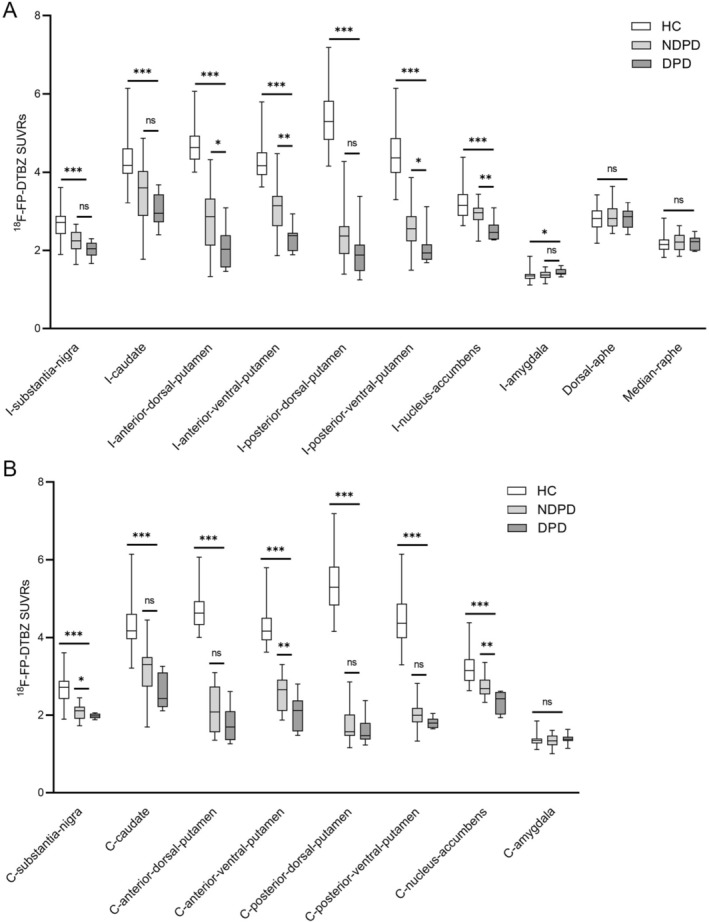
Comparison of subregional ^18^F‐FP‐DTBZ PET standardized uptake value ratios. Differences in monoaminergic disruptions, as assessed by ^18^F‐FP‐DTBZ PET standardized uptake value ratios, in the subregions of the striatum, raphe nucleus, and amygdala between healthy controls (HC), patients with Parkinson's disease and depression (DPD), and those without depression (NDPD) are presented on the ipsilateral (A) and contralateral (B) sides. White bars represent healthy controls, light gray bars represent DPD patients, and dark gray bars represent NDPD patients. I, ipsilateral; C, contralateral. *p < 0.05, **p < 0.01, ***p < 0.001, ns, no significant.

^18^F‐FP‐DTBZ PET SUVRs differed among three groups in the striatal subregions and substantia‐nigra and were substantially decreased in the bilateral substantia‐nigra, caudate, anterior‐dorsal‐putamen, anterior‐ventral‐putamen, posterior‐dorsal‐putamen, posterior‐ventral‐putamen, and nucleus‐accumbens in both NDPD and DPD compared with healthy controls (Figure 2). SUVRs in the DPD group were nominally lower than those in the NDPD group in regions including all putamen subregions except posterior‐dorsal‐putamen on the ipsilateral side, anterior‐ventral‐putamen and substantia‐nigra on the contralateral side, and nucleus‐accumbens on both sides (Figure 2; Table S2). After Tukey's Honestly Significant Difference correction, significant differences persisted in the anterior dorsal and ventral putamen on the ipsilateral side, anterior‐ventral‐putamen on the contralateral side, and both nucleus‐accumbens regions (all corrected *p* < 0.05; Table S2). An increasing ^18^F‐FP‐DTBZ PET uptake trend was observed from healthy controls to NDPD and DPD patients in the dorsal‐raphe and median‐raphe, albeit with no significance. The Parkinson's disease group exhibited significantly increased ^18^F‐FP‐DTBZ PET uptake compared with that of healthy controls in the ipsilateral amygdala (Figure 2A, Table S2; *p* = 0.042). Significant negative correlations were observed between HAMD‐24 scores and ^18^F‐FP‐DTBZ PET SUVRs in the I‐anterior‐ventral‐putamen (*p* = 0.021) and bilateral nucleus‐accumbens regions (I: *p* = 0.016; C: *p* = 0.008). After controlling for duration, significant correlations persisted in the I‐anterior‐ventral‐putamen (*p* = 0.028), C‐anterior‐ventral‐putamen (*p* = 0.038), and bilateral nucleus‐accumbens (I: *p* = 0.022; C: *p* = 0.001; Table 2).

**TABLE 2:** Spearman and partial correlation analyses of regional SUVRs with HAMD‐24 scores in regions of significant monoaminergic deficit in DPD.

Neuropsychological score	^18^F‐FP‐DTBZ PET	*r*	*p*	r*	p*
HAMD‐24	I‐anterior‐ventral‐putamen	−0.451	0.021	−0.440	0.028
	C‐anterior‐ventral‐putamen	−0.360	0.071	−0.417	0.038
	I‐anterior‐dorsal‐putamen	−0.289	0.152	/	0.131
	I‐nucleus‐accumbens	−0.466	0.016	−0.457	0.022
	C‐nucleus‐accumbens	−0.507	0.008	−0.604	0.001

Note: r* and p* values from partial correlation analysis adjusted for disease duration.

Abbreviations: C, contralateral; HAMD‐24, 24‐item Hamilton Depression Rating Scale; I, ipsilateral; SUVRs, standardized uptake value ratios.

### 3.3 Functional Connectivity in the Striatum and Mesolimbic Subregions

#### 3.3.1 Seed‐Based Voxel‐Wise Functional Connectivity

**FIGURE 3:**
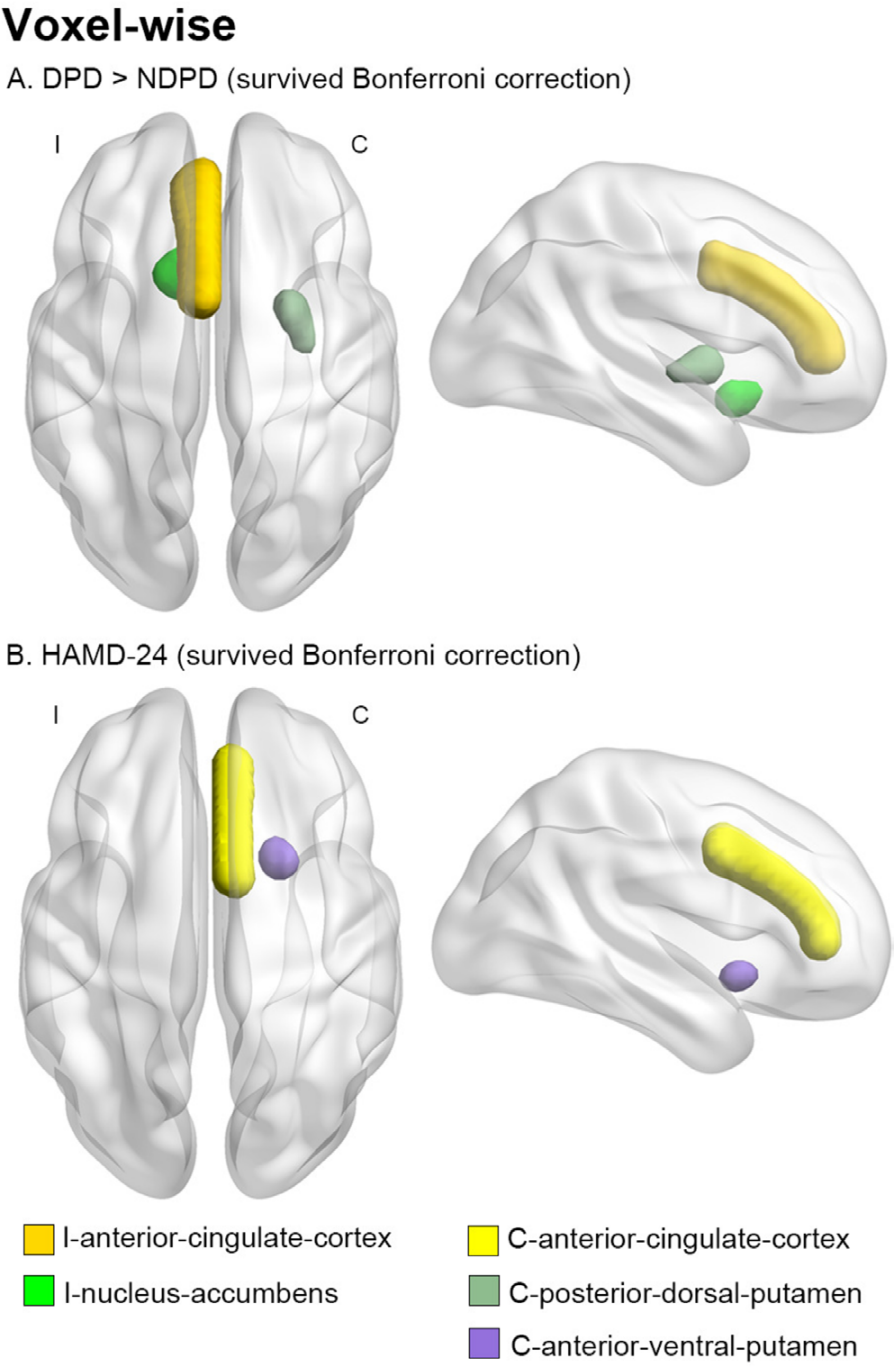
Voxel‐wise functional connectivity differences and correlations in the PET/MRI cohort. (A) Compared to non‐depressed patients (NDPD), those with depression (DPD) showed enhanced functional connectivity between the I‐nucleus‐accumbens and the C‐posterior‐dorsal‐putamen, as well as between the I‐nucleus‐accumbens and the I‐anterior‐cingulate‐cortex. (B) HAMD‐24 scores positively correlated with the functional connectivity between the C‐anterior‐cingulate‐cortex and the C‐anterior‐ventral‐putamen. (Voxel *p* < 0.001, cluster‐level FWE correction *p* < 0.05, > 10 voxels) I, ipsilateral; C, contralateral.

To assess monoaminergic disruption effects on associated functional networks, seed‐based voxel/VOI‐wise functional connectivity analyses were performed. No significant group differences in voxel‐wise functional connectivity emerged across the PET/MRI cohort groups. Compared with NDPD patients, DPD patients exhibited increased functional connectivity between the seed regions of I‐nucleus‐accumbens to I‐anterior‐cingulate‐cortex and C‐posterior‐dorsal‐putamen (Figure 3A; Table S3). HAMD‐24 scores and the seed area from C‐anterior‐ventral‐putamen to C‐anterior‐cingulate‐cortex were positively correlated (Figure 3B; Tables S4 and S5). No significant associations occurred between HAMA and voxel‐wise functional connectivity.

#### 3.3.2 VOI‐Wise Functional Connectivity

**FIGURE 4:**
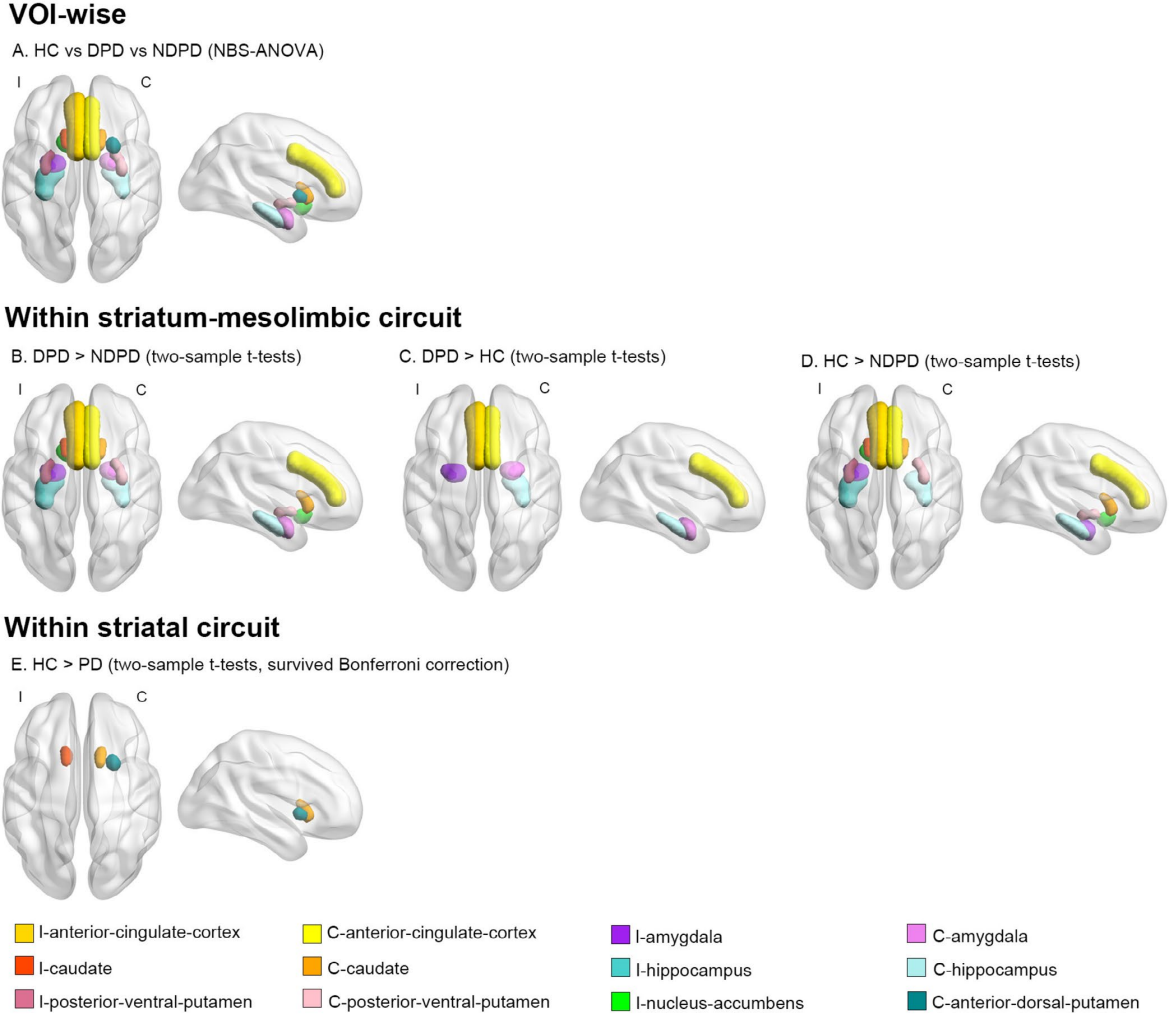
Volume‐of‐interest (VOI) wise functional connectivity differences among healthy controls and Parkinson's disease patients with and without depression in the ComBat‐harmonized data of PET/MRI and MRI validation cohorts. (A) A significant network component differing among healthy controls (HC), Parkinson's disease patients with depression (DPD), and those without depression (NDPD) using omnibus ANOVA analyses. Subsequent pairwise comparisons revealed specific network alterations: (B) Connections within the striatum‐mesolimbic circuit that were significantly stronger in the DPD group than in the NDPD group. (C) Connections significantly stronger in the DPD group than in the HC group. (D) Connections significantly stronger in the HC group than in the NDPD group. (E) A network component within the striatal circuit demonstrating significantly stronger connectivity in the HC group than in the combined PD group (DPD + NDPD), which survived stringent Bonferroni correction for multiple comparisons. I, ipsilateral; C, contralateral.

NBS‐ANOVA analysis (50,000 permutations; edge *p* > 0.01, connected component *p* > 0.05) revealed significant differences in VOI‐wise functional connectivity among the healthy control, NDPD, and DPD groups within the following network components of the striatum‐mesolimbic circuit (Figure 4A): (1) C‐posterior‐ventral‐putamen and bilateral caudate; (2) bilateral anterior‐cingulate‐cortex and I‐caudate; (3) bilateral anterior‐cingulate‐cortex and I‐nucleus‐accumbens; (4) bilateral anterior‐cingulate‐cortex and I‐hippocampus; (5) bilateral anterior‐cingulate‐cortex and C‐amygdala; (6) C‐caudate and bilateral hippocampus; (7) I‐amygdala and C‐caudate; (8) I‐amygdala and C‐anterior‐cingulate‐cortex; (9) I‐hippocampus and I‐posterior‐ventral‐putamen; (10) C‐anterior‐cingulate‐cortex and C‐hippocampus; (11) C‐anterior‐dorsal‐putamen and bilateral caudate.

Subsequent two‐sample *t*‐test analyses further revealed that DPDs exhibited elevated functional connectivity compared with NDPDs in the following circuits involved in emotion regulation (Figure 4B): (1) C‐posterior‐ventral‐putamen and bilateral caudate; (2) bilateral anterior‐cingulate‐cortex and I‐caudate; (3) bilateral anterior‐cingulate‐cortex and I‐nucleus‐accumbens; (4) bilateral anterior‐cingulate‐cortex and I‐hippocampus; (5) C‐caudate and bilateral hippocampus; (6) I‐amygdala and C‐caudate; (7) I‐hippocampus and I‐posterior‐ventral‐putamen. Higher functional connectivity was also noted in healthy controls compared to the NDPD within the above circuits (Figure 4D). No significant difference in functional connectivity was observed between DPDs and healthy controls.

Moreover, functional connectivity was stronger in DPDs relative to NDPDs, as well as healthy controls, between the following regions (Figure 4B,C): (1) bilateral anterior‐cingulate‐cortex and C‐amygdala; (2) I‐amygdala and C‐anterior‐cingulate‐cortex; (3) C‐anterior‐cingulate‐cortex and C‐hippocampus. NDPDs and healthy controls exhibited comparable functional connectivity levels in the regions above.

Within striatal subregions, functional connectivity between the C‐anterior‐dorsal‐putamen and bilateral caudate was stronger in healthy controls than in both DPD and NDPD groups.

When the more stringent Bonferroni correction was applied, only the functional connectivity between C‐anterior‐dorsal‐putamen and bilateral caudate in healthy controls remained significantly stronger than in patients with Parkinson's disease (Figure 4E).

These VOI‐wise functional connectivity findings were derived from ComBat‐harmonized data of PET/MRI and MRI validation cohorts. Analyses repeated independently in the separate PET/MRI and MRI validation cohorts yielded largely similar VOI‐wise functional connectivity patterns (Figure S1).

## 4 Discussion

To the best of our knowledge, this study is the first to combine ^18^F‐FP‐DTBZ PET and rs‐fMRI to identify the striatum‐mesolimbic circuit in patients with Parkinson's disease and depression compared with patients without depression and healthy controls. Relative to both NDPD and HC, DPD patients exhibited the lowest ^18^F‐FP‐DTBZ uptake in the bilateral anterior‐ventral‐putamen and nucleus‐accumbens—regions where binding inversely correlated with depression scores. Furthermore, rs‐fMRI identified elevated but not decreased functional connectivity within the striatum‐mesolimbic circuits in the DPD group, which was not observed in the NDPD group.

Our study provided evidence that PD patients with depressive symptoms had lower monoaminergic binding in most striatal regions, especially in the I‐anterior‐dorsal‐putamen and bilateral anterior‐ventral‐putamen, which underscored the striatum's role in emotion regulation and highlighted the specific involvement of ventral striatal reward and motivation circuits [30, 31]. Similarly, reduced noradrenaline innervation in the left ventral striatum [14] and a pronounced loss of striatal dopamine in Parkinson's disease‐related depression were reported [32], collectively suggesting a link between depression and aggravated monoaminergic degeneration. We also observed inverse correlations between depression severity and VMAT2 distribution in the bilateral anterior‐ventral‐putamen, consistent with studies demonstrating an inverse relationship between affective symptoms and dopaminergic deficits in the ventral striatum [14] and putamen [15]. A recent study using ^18^F‐FP‐DTBZ further confirmed the strong association between reduced striatal VMAT2 and depression severity [33]. Although some studies did not find significant roles for striatal deficits in Parkinson's disease‐related depression [19, 23], confounding factors such as disease duration, motor symptom severity, and antidepressant use may need to be considered. Overall, these results suggest that striatal monoaminergic deficits, particularly in the ventral striatum, play a key role in Parkinson's disease‐related depression.

Meanwhile, we revealed a significant reduction in VMAT2 binding within the nucleus‐accumbens that correlated with depression severity in depressed PD patients. The nucleus‐accumbens receives mixed dopaminergic and glutamatergic inputs from the ventral tegmental area, prefrontal cortex, amygdala, and hippocampus to integrate reward‐related signals and guide motivated behavior [34], which presents as a central hub of the reward circuit. Our finding was consistent with the reported reduction of dopamine transporter availability in the nucleus‐accumbens of patients with major depression [35].

On the other hand, we observed a slight increase in monoaminergic innervation in the amygdala in DPD patients compared to healthy controls. While this finding appears inconsistent with a mixed pattern of increased serotonin transporter [36] and decreased dopamine and noradrenaline binding [14] in the amygdala of PD patients with depression reported before, the discrepancy may stem from small sample size and methodological differences. Future larger‐scale studies are warranted to explore this further.

The raphe nuclei that house approximately one‐third of the brain's serotonergic neurons [37] are intricately linked to reward circuits and mood regulation through extensive serotonergic projections to cortical, subcortical, and midbrain regions [38]. However, we did not find significant monoaminergic abnormalities in the raphe of patients with early‐stage Parkinson's disease and depressive symptoms, which were consistent with previous postmortem evidence [39]. Notably, VMAT2 is predominantly distributed in the terminals of monoaminergic neurons, whereas the raphe consists primarily of cell bodies, and our findings may not specifically reflect serotonergic activity in the raphe nuclei. Thus, the observation of normal or slightly elevated VMAT2 levels in the raphe does not necessarily indicate intact monoaminergic function in patients with Parkinson's disease. Further investigation is warranted to clarify monoaminergic signaling in the raphe nuclei.

Following our initial analysis of monoaminergic deficits in specific striatum‐mesolimbic nuclei, we further investigated potential alterations in functional connectivity across this reward‐processing circuit. Centered on the ventral striatum, nucleus‐accumbens, anterior‐cingulate‐cortex, amygdala, and hippocampus, this network orchestrates motivation, hedonic experiences, and contextual learning via coordinated dopaminergic and serotonergic signaling [7]. We observed greater functional connectivity in DPD versus NDPD patients between the seed regions of I‐nucleus‐accumbens to I‐anterior‐cingulate‐cortex and C‐posterior‐dorsal‐putamen. VOI‐wise functional connectivity analyses revealed a trend toward heightened connectivity within the emotion‐related striatum‐mesolimbic circuit in DPD versus NDPD patients at an uncorrected threshold. Notably, similar patterns were observed in the independent MRI validation cohort and the ComBat‐harmonized integrated dataset, indicating certain replicability of the results. Supporting our findings, Dan et al. also reported heightened functional connectivity within the striatum‐mesolimbic loop in Parkinson's disease, which correlated with greater depression severity [40]. Increased functional connectivity between the left amygdala and limbic regions has been reported in DPD patients compared to both NDPD patients and healthy controls [41]. Similarly, some studies have demonstrated elevated degree centrality in the bilateral anterior‐cingulate‐cortex, alongside enhanced seed‐based functional connectivity between the bilateral anterior‐cingulate‐cortex and prefrontal cortex in DPD groups relative to NDPD and control groups [42]. A recent study also noted increased hub distribution within the limbic‐cortico‐basal ganglia circuit in DPD patients compared with that of the NDPD and healthy controls [22].

The mechanism of significant monoaminergic depletion alongside increased functional connectivity within the striatum‐mesolimbic circuits in the DPD patients was unknown. While some reports attribute such connectivity enhancements to compensatory mechanisms [43], others interpret heightened connectivity as reflections of dysfunctional network integration and aberrant neural signaling [44]. Considering that hyperconnectivity may compensate for disrupted pathways, as observed in late‐life depression [45], we speculate that the observed early‐stage hyperconnectivity within the striatum‐mesolimbic circuit may represent a dual response aimed at counteracting initial monoaminergic deficits (mainly dopaminergic) and mitigating depressive pathology.

This study possesses some limitations. First, brain regions analyzed for PET (substantia nigra, striatum, amygdala, and raphe nuclei) and rs‐fMRI (anterior‐cingulate‐cortex, striatum, amygdala, and hippocampus) did not completely overlap. This was partly due to the poor cortical VMAT2 binding signal, which hindered reliable PET measurements in the hippocampus and anterior‐cingulate‐cortex, combined with the small volume and susceptibility to signal contamination of the substantia nigra, which compromised its assessment in fMRI [46]. Second, although we carefully screened participants and excluded those with clinically significant anxiety, potential confounding effects of anxiety in depression‐related findings cannot be entirely excluded. Third, we acknowledge that the initial sample size was limited, largely due to the challenges of recruiting early‐stage patients with Parkinson's disease and depression, and who had not received antidepressant treatment. To proactively address this constraint and improve statistical reliability, we expanded our recruitment for an independent MRI validation cohort. Nonetheless, further validation in larger, multi‐center cohorts remains an important future goal.

## 5 Conclusions

Alterations in monoaminergic neurotransmitter expression are likely associated with abnormal functional connectivity within the striatum‐mesolimbic circuit, which may reflect disrupted molecular signal transmission and impaired neural network connectivity in patients with early Parkinson's disease and depression. Collectively, our results position the monoaminergic system as a potential target for treating Parkinson's disease‐related depression, with the aim of restoring both chemical balance and neural connectivity.

## Author Contributions

S.‐Y.L., C.Z., and P.C.: study concept, design, and manuscript revision for important intellectual content. S.‐Y.L. and X.‐L.L.: clinical data collection. S.‐Y.L. and O.B.: imaging data analysis. H.‐W.Q., G.D.T., C.Z., and J.L.: imaging data collection. B.‐L.X. and X.‐L.L.: manuscript writing. All authors read and approved the final manuscript.

## Funding

This study was supported by grants from the National Natural Science Foundation of China (81901285), the Beijing Nova Program (20240484504), and the Ministry of Science and Technology of China (2021YFC2501200).

## Ethics Statement

This study was approved by the Ethics Committee of Xuanwu Hospital (approval number: 2019[014] and 2022[047]).

## Consent

Written informed consent was obtained from participants.

## Conflicts of Interest

The authors declare no conflicts of interest.

## Supporting information


**Table S1:** Clinical and demographic data of MRI validation cohort.
**Table S2:** Comparison of Subregional SUVRs.
**Table S3:** Difference of functional connectivity for DPD and NDPD patients.
**Table S4:** Voxel‐wise multiple regression analysis of functional connectivity for HAMD‐24 scores in PD patients.
**Table S5:** Voxel‐wise multiple regression analysis of functional connectivity for HAMD‐24 scores in PD patients using age, sex and MDS‐UPDRS III as co‐variants.
**Figure S1:** Volume‐of‐interest (VOI) wise functional connectivity patterns in PET/MRI cohort and MRI validation cohort. (A–C) Network components showing significant differences in functional connectivity within the striatum‐mesolimbic circuit from the PET/MRI cohort, as identified by two‐sample *t*‐tests: (A) Connections stronger in depressed Parkinson's disease (DPD) patients than in Non‐depressed Parkinson's disease (NDPD) patients; (B) Connections stronger in DPD patients than in Healthy Controls (HC); (C) Connections stronger in HC than in NDPD patients. (D) The only connection within the striatum‐mesolimbic circuit that remained significantly stronger in DPD than in NDPD patients after stringent Bonferroni correction for multiple comparisons. (E) A network component within the striatal circuit where HC showed stronger functional connectivity than NDPD patients in two‐sample *t*‐tests. (F) Network components showing stronger functional connectivity in DPD than in NDPD patients within the MRI validation cohort, as identified by two‐sample *t*‐tests (results did not survive Bonferroni correction). I, ipsilateral; C, contralateral.

## Data Availability

The datasets analyzed during the current study are not publicly available due to privacy/ethical restrictions but are available from the corresponding author on reasonable request.
