# Supplementary material for: Monoamine Loss and Functional Connectivity Alterations in Patients With Parkinson's Disease and Depression
Source: CNS Neurosci Ther. 2025 Dec 25;31(12):e70705. doi: 10.1002/cns.70705 (PMC12741543; doi:10.1002/cns.70705)

**Supplementary Results**

**VOI-wise Functional Connectivity Patterns in PET/MRI Cohort**

NBS analysis (50,000 permutation tests, edge P>0.01, connected P>0.05) detected no significant differences among healthy controls (HC), NDPD, and DPD groups. Subsequent between-group t-tests identified specific functional connectivity alterations for the following region pairs:(1) Compared to NDPD patients, DPD patients exhibited elevated VOI-wise functional connectivity between: (i) bilateral anterior-cingulate-cortex and bilateral caudate; (ii) bilateral anterior-cingulate-cortex and contralateral-anterior-ventral-putamen; (iii) contralateral-caudate and ipsilateral-nucleus-accumbens; (iv) contralateral-caudate and ipsilateral-anterior-ventral-putamen; (v) contralateral-caudate and ipsilateral-hippocampus; (vi) ipsilateral-posterior-ventral-putamen and contralateral-amygdala. No significant HC-DPD differences were observed in these pathways. (2) Relative to HC, the DPD group showed stronger functional connectivity between: (i) ipsilateral-caudate and contralateral-anterior-cingulate-cortex; and (ii) ipsilateral-posterior-ventral-putamen and contralateral-hippocampus. (3) Within emotion-regulation-related striatal subregions and extra-striatal regions, HC demonstrated enhanced functional connectivity versus NDPD in the following pairs: (i) ipsilateral-nucleus-accumbens and contralateral-nucleus-accumbens; (ii) ipsilateral-nucleus-accumbens and contralateral-posterior-ventral-putamen; (iii) ipsilateral-nucleus-accumbens and contralateral-caudate; (iv) ipsilateral-hippocampus and ipsilateral-posterior-ventral-putamen; (v) bilateral anterior-cingulate-cortex and ipsilateral-hippocampus. Within striatal subregions, HC exhibited stronger functional connectivity than NDPD patients between contralateral-anterior-dorsal-putamen and bilateral caudate. Additionally, two-sample t-tests indicated a gradient of functional connectivity strength between the ipsilateral-nucleus-accumbens and bilateral anterior-cingulate-cortex across groups: DPD > HC > NDPD. However, after applying the Bonferroni correction for multiple comparisons, only the functional connectivity between ipsilateral-nucleus-accumbens and bilateral anterior-cingulate-cortex in DPD patients remained significantly stronger than that in NDPD patients.

**Functional Connectivity Patterns in MRI Validation Cohort**

To validate the reproducibility of our primary findings, we conducted replication analyses in an extended MRI cohort comprising 20 NDPD and 9 DPD patients using identical preprocessing pipelines and statistical frameworks. Demographic characteristics (age, sex, education, disease duration) did not differ between groups. Seed-based voxel-wise analyses revealed no significant group differences in functional connectivity patterns, nor associations between depression/anxiety severity scores and functional connectivity metrics in PD patients. On the other hand, VOI-wise functional connectivity analyses based on Network-based analysis (50,000 permutation tests, edge P>0.01, connected P>0.05) showed significantly enhanced functional connectivity in DPD versus NDPD patients across specific region pairs: (i) contralateral-caudate and contralateral-posterior-dorsal-putamen; (ii) contralateral-caudate and contralateral-posterior-ventral-putamen; (iii) contralateral-caudate and ipsilateral-amygdala; (iv) contralateral-caudate and bilateral hippocampus; (v) ipsilateral-caudate and ipsilateral-hippocampus; (vi) ipsilateral-caudate and ipsilateral-amygdala; (vii) contralateral-posterior-dorsal-putamen and contralateral-hippocampus; (viii) ipsilateral-amygdala and ipsilateral-hippocampus; (ix) contralateral-anterior-cingulate-cortex and bilateral hippocampus; (x) bilateral amygdala and bilateral anterior-cingulate-cortex. Also, two-sample t-tests independently confirmed the network-level findings. However, no functional connectivity within the VOI-wise functional connectivity remained statistically significant after Bonferroni correction for multiple comparisons.

**Supplementary Table 1.** Clinical and demographic data of MRI validation cohort

|  | **NDPD (N = 20)** | **DPD (N = 9)** | **P** |
| --- | --- | --- | --- |
| **Age (y)** | 55.8 ± 11.7  (38.4 - 71.8) | 56.4 ± 10.1  (38.9 - 70.5) | 0.896^c^ |
| **Gender** | 8F/12M | 3F/6M | 0.737^d^ |
| **Disease Duration (m)** | 21.70 ± 12.35  (2-42) | 24.67 ± 17.28  (5 - 53) | 0.736 ^c^ |
| **H-Y Scale** | 1.58 ± 0.47  (1.0 - 2.0) | 1.72 ± 0.51  (1.0 - 2.5) | 0.475^d^ |
| **MDS-UPDRS III** | 22.6 ± 9.96  (5 - 35) | 28.4 ± 14.53  (11 - 58) | 0.216^c^ |
| **MoCA** | 25.80 ± 2.75  (21 - 30) | 24.78 ± 4.52  (14 - 29) | 0.456^c^ |
| **HAMD-17** | 2.00 ± 1.75  (0 - 6) | 10.00 ± 3.57  (7 - 17) | <0.001^d^ |
| **HAMA** | 2.15 ± 1.60  (0 - 5) | 4.78 ± 5.07  (0 - 14) | 0.336^d^ |
| **RBDQ-HK** | 9.60 ± 9.11  (0 - 29) | 20.00 ± 17.77  (0 - 49) | 0.185^d^ |
| **PDQ-39** | 12.35 ± 9.21  (0 - 33) | 22.22 ± 22.06  (3 - 76) | 0.220^d^ |

Abbreviations: DPD, patients with Parkinson’s disease and depression; NDPD, patients with Parkinson’s disease and without depression; H-Y Scale, Hoehn-Yahr Scale; MDS-UPDRS III, the third part of the MDS-Unified Parkinson’s Disease Rating Scale; MoCA, Montreal Cognitive Assessment; HAMA, Hamilton Anxiety Scale; HAMD-17, 17-item Hamilton Depression Rating Scale; RBDQ-HK, Rapid Eye Movement Sleep Behavior Disorder Questionnaire-Hong Kong; PDQ-39, Parkinson's Disease Questionnaire.

Data are presented as mean ± SD, with ranges in parentheses.

^c^P value from Student’s t-test between the NDPD and DPD groups.

^d^P value from Mann-Whitney U test between the NDPD and DPD groups.

**Supplementary Table 2.** Comparison of Subregional SUVRs

|  | **HC** | **DPD** | **NDPD** | **P** | | | **DPD** | **NDPD** | **P** | | |
| --- | --- | --- | --- | --- | --- | --- | --- | --- | --- | --- | --- |
|  |  | **Ipsilateral** | | **ANOVA** | **DPD vs NDPD** | **DPD vs NDPD** | **Contralateral** | | **ANOVA** | **DPD vs NDPD** | **DPD vs NDPD** |
| **substantia-nigra** | 2.70±0.34 | 2.02±0.21 | 2.22±0.29 | <0.001^a^ | 0.084^c^ | 0.273^e^ | 1.97±0.06 | 2.10±0.20 | <0.001^a^ | 0.030^c^ | 0.553^e^ |
| **caudate** | 4.29±0.56 | 3.03±0.42 | 3.45±0.85 | <0.001^b^ | 0.194^c^ | 0.290^e^ | 2.59±0.46 | 3.12±0.73 | <0.001^b^ | 0.076^c^ | 0.121^e^ |
| **anterior-dorsal-putamen** | 4.75±0.58 | 2.06±0.55 | 2.83±0.85 | <0.001^b^ | 0.028^c^ | 0.026^e^ | 1.76±0.46 | 2.15±0.57 | <0.001^b^ | 0.108^c^ | 0.248^e^ |
| **anterior-ventral-putamen** | 4.26±0.49 | 2.30±0.34 | 3.08±0.65 | <0.001^b^ | 0.004^c^ | 0.004^e^ | 2.07±0.45 | 2.59±0.43 | <0.001^b^ | 0.009^c^ | 0.027^e^ |
| **posterior-dorsal-putamen** | 5.35±0.72 | 1.94±0.66 | 2.43±0.82 | <0.001^b^ | 0.085^d^ | 0.275^e^ | 1.59±0.37 | 1.74±0.43 | <0.001^a^ | 0.406^c^ | 0.826^e^ |
| **posterior-ventral-putamen** | 4.46±0.68 | 2.07±0.46 | 2.54±0.63 | <0.001^b^ | 0.030^d^ | 0.192^e^ | 1.8±0.14 | 2.00±0.36 | <0.001^a^ | 0.154^c^ | 0.677^e^ |
| **nucleus-accumbens** | 3.23±0.42 | 2.52±0.28 | 2.93±0.30 | <0.001^b^ | 0.003^c^ | 0.030^e^ | 2.33±0.29 | 2.72±0.27 | <0.001^b^ | 0.003^c^ | 0.036^e^ |
| **amygdala** | 1.34±0.13 | 1.44±0.09 | 1.37±0.11 | 0.042^b^ | 0.113^c^ | 0.309^e^ | 1.38±0.14 | 1.34±0.16 | 0.612^b^ | / | **/** |
| **dorsal-raphe** | 2.79±0.30 | 2.83±0.27 | 2.87±0.31 | 0.689^a^ | 0.722^c^ | 0.933^e^ | / | / | / | / | / |
| **medial-raphe** | 2.19±0.24 | 2.19±0.19 | 2.21±0.24 | 0.888^b^ | 0.908^c^ | 0.993^e^ | / | / | / | / | / |

Abbreviations: SUVRs, standardized uptake value ratios; HC, healthy controls; DPD, patients with Parkinson’s disease and depression; NDPD, patients with Parkinson’s disease and without depression; ANOVA, one-way analysis of variance.

^a^P value from one-way ANOVA test among three groups of HC, NDPD, and DPD.

^b^P value from the Kruskal–Wallis test among the HC, NDPD, and DPD groups.

^c^P value from Student’s t-test between the NDPD and DPD groups.

^d^P value from Mann-Whitney U test between the NDPD and DPD groups.

^e^P value after Turkey’s Honestly Significant Difference correction for the NDPD vs. DPD comparison.

**Supplementary Table 3.** Difference of functional connectivity for DPD and NDPD patients

| **Seed area** | **I/C** | **Size** | **Brain Region** | **Peak MNI coordinates** | | | **T value** |
| --- | --- | --- | --- | --- | --- | --- | --- |
|  |  |  |  | **x** | **y** | **z** |  |
| **DPD>NDPD** | | | | | | | |
| **I-nucleus-accumbens** | I | 45 | anterior-cingulate- cortex | -3 | 45 | 12 | 4.8087^*^ |
| **I-nucleus-accumbens** | C | 10 | posterior-dorsal-putamen | 30 | -12 | 0 | 5.1828^*^ |

* Voxel P<0.001, cluster P <0.05, KE >10, FWE corrected

Abbreviations: DPD, patients with Parkinson’s disease and depression; NDPD, patients with Parkinson’s disease and without depression; MNI, Montreal Neuroscience Institute; I, ipsilateral; C, contralateral.

Age, sex, education, and duration were used as co-variants.

**Supplementary** **Table 4.** Voxel-wise multiple regression analysis of functional connectivity for HAMD-24 scores in PD patients

| **Seed area** | **I/C** | **size** | **Brain Region** | **Peak MNI coordinates** | | | **T value** |
| --- | --- | --- | --- | --- | --- | --- | --- |
|  |  |  |  | **x** | **y** | **z** |  |
| **HAMD-24** | | | | | | | |
| **C-anterior-ventral-putamen** | C | 12 | anterior-cingulate- cortex | 12 | 21 | 30 | 8.3550* |

* Voxel P<0.001, cluster P <0.05, KE >10, FWE corrected

Abbreviations: MNI, Montreal Neuroscience Institute; HAMD-24, 24-item Hamilton Depression Rating Scale; I, ipsilateral; C, contralateral.

Age, sex and disease duration were used as co-variants.

**Supplementary** **Table 5.** Voxel-wise multiple regression analysis of functional connectivity for HAMD-24 scores in PD patients using age, sex and MDS-UPDRS III as co-variants

| **Seed area** | **I/C** | **size** | **Brain Region** | **Peak MNI coordinates** | | | **T value** |
| --- | --- | --- | --- | --- | --- | --- | --- |
|  |  |  |  | **x** | **y** | **z** |  |
| **HAMD-24** | | | | | | | |
| **C-anterior-ventral-putamen** | C | 10 | anterior-cingulate- cortex | 12 | 21 | 30 | 8.4297^*^ |

* Voxel P<0.001, cluster P <0.05, KE >10, FWE corrected

Abbreviations: MNI, Montreal Neuroscience Institute; HAMD-24, 24-item Hamilton Depression Rating Scale; I, ipsilateral; C, contralateral.

Age, sex and MDS-UPDRS III were used as co-variants.

**Supplementary** **Figure 1. Volume-of-interest (VOI) wise functional connectivity patterns in PET/MRI cohort and MRI validation cohort.**

(A-C) Network components showing significant differences in functional connectivity within the striatum-mesolimbic circuit from the PET/MRI cohort, as identified by two-sample t-tests: (A) Connections stronger in depressed Parkinson's disease (DPD) patients than in Non-depressed Parkinson's disease (NDPD) patients; (B) Connections stronger in DPD patients than in Healthy Controls (HC); (C) Connections stronger in HC than in NDPD patients. (D) The only connection within the striatum-mesolimbic circuit that remained significantly stronger in DPD than in NDPD patients after stringent Bonferroni correction for multiple comparisons. (E) A network component within the striatal circuit where HC showed stronger functional connectivity than NDPD patients in two-sample t-tests. (F) Network components showing stronger functional connectivity in DPD than in NDPD patients within the MRI validation cohort, as identified by two-sample t-tests (results did not survive Bonferroni correction). I, ipsilateral; C, contralateral.


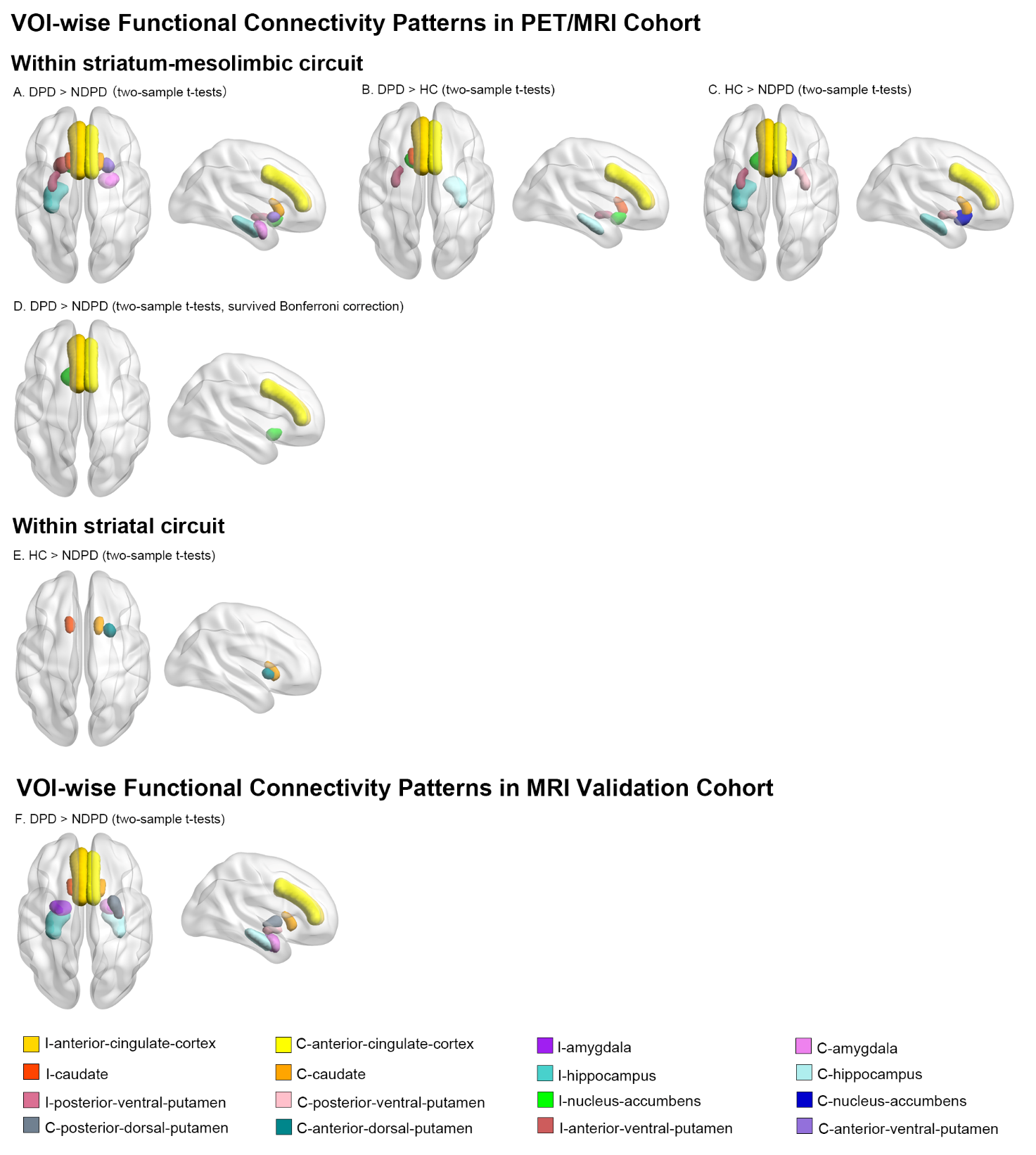

Supplement: Supplementary file 1 — Table S1: Clinical and demographic data of MRI validation cohort. Table S2: Comparison of Subregional SUVRs. Table S3: Difference of functional connectivity for DPD and NDPD patients. Table S4: Voxel‐wise multiple regression analysis of functional connectivity for HAMD‐24 scores in PD patients. Table S5: Voxel‐wise multiple regression analysis of functional connectivity for HAMD‐24 scores in PD patients using age, sex and MDS‐UPDRS III as co‐variants. Figure S1: Volume‐of‐interest (VOI) wise functional connectivity patterns in PET/MRI cohort and MRI validation cohort. (A–C) Network components showing significant differences in functional connectivity within the striatum‐mesolimbic circuit from the PET/MRI cohort, as identified by two‐sample t‐tests: (A) Connections stronger in depressed Parkinson's disease (DPD) patients than in Non‐depressed Parkinson's disease (NDPD) patients; (B) Connections stronger in DPD patients than in Healthy Controls (HC); (C) Connections stronger in HC than in NDPD patients. (D) The only connection within the striatum‐mesolimbic circuit that remained significantly stronger in DPD than in NDPD patients after stringent Bonferroni correction for multiple comparisons. (E) A network component within the striatal circuit where HC showed stronger functional connectivity than NDPD patients in two‐sample t‐tests. (F) Network components showing stronger functional connectivity in DPD than in NDPD patients within the MRI validation cohort, as identified by two‐sample t‐tests (results did not survive Bonferroni correction). I, ipsilateral; C, contralateral. [file CNS-31-e70705-s001.docx]
